# “Not all groups come together, but this one just clicks”: Ten Tips for Sustaining an Engagement Panel

**DOI:** 10.1007/s11606-021-06985-1

**Published:** 2022-03-29

**Authors:** Agnes C. Jensen, Erin C. Amundson, Cheryl Brown, James Cochrane, David Daly, Rosie Glenn, Kaydance Hope, Jeanette Hawkins, Joshua Wilder Oakley, Elijah Sacra, Janet Walker, William C Westmoreland Sr

**Affiliations:** 1 Center for Care Delivery and Outcomes Research (CCDOR, Code 152), Minneapolis VA Health Care System, 1 Veterans Drive, Minneapolis, MN 55417 USA; 2 VOICE Veteran Engagement Panel, Minneapolis, Minnesota USA

## Abstract

In 2017, ten veteran patients with the shared experience of living with chronic pain united to form a Veteran Engagement Panel (VEP) to support the Patient-Centered Outcomes Research Institute® (PCORI®)funded Veterans Pain Care Organizational Improvement Comparative Effectiveness (VOICE) Study. The study, conducted at ten Veterans Affairs (VA) sites, compares two team-based approaches to improve pain management and reduce potential harms of opioid therapy. The panel shares ten best practices for sustaining a successful engagement partnership.

In 2017, ten veteran patients with the shared experience of living with chronic pain united to form a Veteran Engagement Panel (VEP) to support the Patient-Centered Outcomes Research Institute® (PCORI®)funded Veterans Pain Care Organizational Improvement Comparative Effectiveness (VOICE) Study^1^. The study, conducted at ten Veterans Affairs (VA) sites, compares two team-based approaches to improve pain management and reduce potential harms of opioid therapy. Along with our two VOICE engagement liaisons, we share ten thoughts on sustaining a successful engagement partnership throughout the life of a project. Our quotations below were thoughts shared during sessions co-authoring this narrative and during semi-annual evaluations of our VEP experience.

1. *Build, then continuously cultivate, a climate of respect*. The VEP was created via a multi-step process using a study engagement committee that included one veteran/staffer and three investigators. Each study site was provided partner position requirements (living with chronic pain and willingness to share perspectives within a group, respect confidentiality, commit to monthly meetings and communicate via email/telephone). Site researchers were asked to share the opportunity with local clinicians/researchers or personally connect with potential partners. Potential partners were asked to submit a statement of interest (self-identifying age, race/ethnicity, and gender) and interviewed via telephone by two committee members. Soliciting volunteer engagement partners is common, however individual volunteers may have personal agendas.^2^ To address this issue, during the interview potential partners were asked to share motivation for participating, collaboration and military experience, services received at VA, and potential barriers to participation. The interview was framed as a two-way conversation, giving partners the opportunity to discuss the panel, researchers’ collaboration style, and mutual expectations. After interviews, the engagement committee reviewed applicants and invited five men and five women to the panel: each with diverse chronic pain experiences, life demands, military history, and age/race. Although our VEP had not previously worked together, during our initial in-person orientation, we immediately connected via the bonds of living with chronic pain and our previous service. The VEP and research team regularly address each other by first names, to eliminate barriers based on previous military rank/hierarchy or academic titles. Our shared experiences and desire to help fellow veterans created an initial foundation of respect. We continuously cultivate that respectful climate by allowing equal time to share input during meetings, valuing differing opinions, and sharing opportunities to represent the group. “The group is very positive and very comfortable. I’ve never been in a group that gets along so well and supports each other this much.” "Feeling respected in the group allows for honest, open dialogue.” We seek to understand each other's viewpoints.
2. *Dedicate time to relationship building—it provides unexpected dividends*. At orientation, we devoted time to get acquainted and share stories. We continue to start each meeting with a check-in. Icebreaker games create bonds and reveal hidden talents. We learned one VEP member has experience in graphic design and another in marketing; both give excellent advice when reviewing materials. Several of us are caregivers to others while managing our own pain; we keep that in mind when considering participant study burden. Our veteran/staffer also promotes cross-connections. “Having a veteran as a facilitator is helpful to develop buy-in with the group and make sure there is a cultural competence around veteran needs.”
3. *Communicate consistently*. Two consistent VOICE staff serve as VEP liaisons and provide reliable, clear communication. At each meeting, they refer to study milestones, feedback from PCORI®, and study investigators. The VEP gives input on meeting times and frequency (currently bi-monthly). Between meetings, staff send updates of study progress, engagement opportunities, and future agendas. “This is the best communication I’ve had in any business or interactions I have. This is the gold standard.”
4. *Be accountable to the panel for how feedback is being used*. At each meeting, we review prior meeting discussions and the impact of our advice. “The continual feedback that has been given by the VEP has significantly been seen in the products and the process. This point is so important as you are making the most out of the VEP’s time and input.”
5. *Create a culture of transparency*. VEP members want to be asked to problem solve, not just to be informed the project is going well. The VOICE study, like many trials (particularly during COVID-19), experienced difficulties with recruitment*.* “I liked that we were *asked* for our help. It made me realize they really do *need* our help.” Researchers should share both successes *and* challenges and solicit problem-solving ideas from engagement partners. We want to help. “The only thing I’m looking for is to get more involved.”
6. *Remain flexible and responsive to change*. Throughout the project, our VEP supported adapting to changing circumstances. For example, the study’s adoption of video technology. In early 2018, video visits were limited throughout the VA. Our VEP encouraged the study to adopt video visits as a way to allow participation by rural pain patients or those without transportation. We helped create talking points to communicate the advantages of video appointments, and by mid-2018 video was integrated as an option at multiple sites. When COVID-19 emerged, experience from 18 months of video use allowed VOICE to transition completely to video and telephone visits. However, the shift completely away from in-person contact sparked fresh dialogue. To address the burden COVID-19 places on chronic pain patients seeking care, we suggested creating a list of virtual resources to share with patients unable to access in-person non-medication pain management options. To create personal connections virtually, we suggested all VOICE clinicians mail short biographies (with a photograph) to each patient before a phone or video appointment. Both these suggestions were implemented by the study team. Our panel also adapted and now meets via video.
7. *Regularly evaluate the panel experience*. Our VEP liaisons encourage giving feedback after each meeting and conduct formal evaluations semi-annually. A 2019 review cites regular evaluation of engagement activity as a best practice, but notes a validated tool is not regularly in use.^3^ Our team has used multiple evaluation methods (one-on-one interviews by a study team member, interviews by an independent evaluator, and online survey). VEP feedback has been implemented to improve meeting processes. One VEP member suggested regularly changing the meeting speaking order as members often expand on thoughts of the person before them; rotating the order would allow for different combinations and perspectives. “I’m pleased that we went with the rotation of the round-robin format, which I think is working out great.”
8. *Leverage engagement participation as a conduit to learning and personal growth*. As one member said, “we’ve grown individually and collectively.” Our VOICE engagement liaisons share opportunities to participate in local research events and regional and national conferences. New opportunities are presented to our panel collectively, to provide equal access for participation. Three members are now “inaugural members” of new VA engagement panels. One member started an MPH program; another completed his doctorate and shared, “Being a member of this supportive group of veterans, aided in my journey and enhanced both my abilities and drive for helping to improve the endeavors of researchers studying care for our fellow veterans experiencing chronic pain. Our VEP brought together generations of military service, all with the same mindset: to bring better care and treatment for current and future veterans seeking care.”
9. *Appreciate that life changes may impact engagement ability, but leave the door open to return*. The VOICE study is a multi-year project. Multiple members have taken “leaves of absence” for health challenges, new jobs, educational opportunities, or unexpected geographic moves. One member shared, “I want to thank the staff and VEP members for not giving up on me.” As a group, we overwhelmingly decided to keep an open-ended membership policy, to allow for a return to the panel when and if able. Life experiences during these absences enrich panel members’ input and conversation upon return.
10. *Recognize the benefits of engagement are felt at multiple levels*. The role of engagement partners is to provide the perspective of our fellow patients and give voice to their concerns. One member describes the role by simply stating, “I help veterans; that’s what I do.” Another stated, “As service to others, we are contributing to creating truly patient-centered care.” Participating on the panel has also paid unexpected personal benefits as we manage our own chronic pain. Our members describe this benefit in the following ways: “I like being able to help other veterans, but it is more than that. I find that I am learning about treatments that I did not know about.” “Knowing and being a part of such a study, provides the knowledge to engage with my primary care provider, in a more well-informed manner for explaining my needs for medical care.”

The VEP allows us to continue serving and we strive to keep improving our partnership. We hope these ten steps will help your engagement panel begin and sustain a relationship like ours.
